# Investigation on the Influence of Chain Extenders on the Performance of One-Component Moisture-Curable Polyurethane Adhesives

**DOI:** 10.3390/polym9050184

**Published:** 2017-05-21

**Authors:** Chen Tan, Teija Tirri, Carl-Eric Wilen

**Affiliations:** Center of Excellence for Functional Materials (FUNMAT), Faculty of Science and Engineering, Laboratory of Polymer Technology, Åbo Akademi University, Biskopsgatan 8, 20500 Turku, Finland; ctan@abo.fi (C.T.); teija.tirri@abo.fi (T.T.)

**Keywords:** adhesive, isocyanate-terminated urethane prepolymers, segmented polyurethane, soft and hard segments

## Abstract

In this work, a number of chain extended moisture-curable urethane prepolymers were synthesized in order to develop isocyanate terminated urethane prepolymer formulations that would simultaneously display both high adhesive strength and low viscosity. Proton nuclear magnetic resonance spectroscopy (^1^H-NMR), size exclusion chromatography (SEC), differential scanning calorimetry (DSC), and Brookfield viscometry were utilized for characterizing the prepared urethane prepolymers. In addition, the adhesion strength of the cured prepolymers was determined by tensile shear strength test according to the DIN EN (Deutsches Institut für Normung, the German Institute for Standardization) 1465 standard. Especially, the role of different types of linear (butanediol, pentanediol) and branched chain extenders (dipropyleneglycol (di-PPG), tripropyleneglycol (tri-PPG) and the influence of their dosage on the degree of microphase separation between hard segments (HS) and soft segments (SS) in urethane prepolymers were studied. Furthermore, the benefits of utilizing either a one-step versus a two-step polymerization process were investigated. The results revealed that the extent of phase separation of different urethane prepolymers was dependent on the extent of hydrogen bonding interactions which was extensively studied by attenuated total reflectance infrared spectroscopy (ATR-FTIR). The incorporation of branched chain extenders (di-PPG and tri-PPG) did not result in notable phase separation between hard segments and soft segments, while linear chain extenders (pentanediol and butanediol) readily promoted phase separation. The degree of phase separation was particularly pronounced for butanediol, and when the linear chain extender ratio was higher than or equal to 0.74. Compared with a two-stage process, one-stage process produced more randomly distributed polymer chains with highly dispersed hard segments. Thus, urethane prepolymers exhibiting strong adhesive strength with simultaneously low viscosity were successfully developed by systematic adjustment of structural parameters.

## 1. Introduction

**Figure 1:**
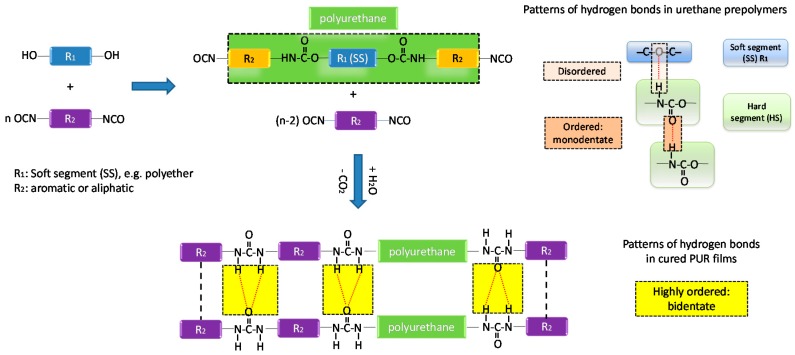
General synthesis and curing processes of moisture-curable urethane prepolymers (MCPUs) and the patterns of hydrogen bonds in their prepolymer and cured films.

Isocyanate-terminated urethane prepolymers are curable by ambient moisture, and widely used as one-component adhesives, due to their effective wetting properties, low viscosity, fast curing speed, ease of handling, toughness, and good water and chemical resistance [1,2,3,4,5,6,7,8]. The unique characteristics of moisture-curable urethane prepolymers (MCPUs) stem from their specific dual microphase structure (the thermodynamic incompatibility of soft segments and hard segments) and inter- and intraphase interactions (mainly hydrogen bonding) in their prepolymer and cured forms, as shown in Figure 1. The soft segment (SS) is made up of polyols that impact flexibility and softness, and hard segments (HS) derive from isocyanates that in turn provide cohesive strength to the polymer matrix as reinforcement units [9,10]. The curing of urethane prepolymers introduces strong urea linkages that can organize into bidentate form, which can further reinforce the material. In general, the cohesive bond strength of MCPU adhesives is largely controlled by the degree of monodentate and bidentate hydrogen bonds [11].

In general, as opposed to the in situ highly cross-linked rigid adhesives such as melamine-formaldehyde (MF) or phenol-resorcinol-formaldehyde (PFR), MCPU adhesives have become a prominent class of flexible adhesives [6,12]. The cohesive strength of MCPUs can be enhanced by adjusting the extent of microphase separation between hard and soft segments, for instance, via the incorporation of short chain diols called chain extenders [2]. The incorporation of chain extenders endows greater immiscibility between non-polar soft and highly polar hard segments, and allows hard segments to phase segregate into hard domains via hydrogen bonding interactions. Hence, the physical crosslinking structure of HS domains reinforces the soft segment matrix, and subsequently promotes mechanical strength and physical properties [13]. However, on the other hand, the complete phase separation and substantial hydrogen bonding interactions give rise to high viscosity, and consequently result in poor substrate wetting.

Therefore, a good balance between phase separation and phase mixing of hard and soft segments is vital in achieving outstanding bond performance for MCPU based adhesives [11,14,15,16]. Hydrogen bonding interactions between hard segment domains induce phase separation, whereas hydrogen bonds between hard and soft segments favor phase mixing [11,14]. Moreover, the miscibility between hard and soft segments can be tuned by either increasing the crystallinity of soft segments by using polyols having carbonate groups along the polymer backbone, or by decreasing the crystallinity of hard segments by using branched chain extenders, or by decreasing their dosage [13]. From an economic viewpoint, the latter method provides the possibility for developing an inexpensive MCPU adhesive exhibiting simultaneously high cohesive strength and low viscosity.

Our objective was to explore the possibility of synthesizing chain-extended MCPU adhesives that have low viscosity and simultaneously exhibit high adhesive strength upon curing. Even though a number of research studies suggest that the properties of MCPUs are largely dependent on the chemical structure of polyols and isocyanates, high performance was usually only realized by the adverse increase in cost, which greatly limits their industrial utilization [1,2,3,4,8,9,13,17,18]. In this work, the authors attempt to improve the cohesive strength of MCPUs made from cheap raw materials polypropylene glycol (PPG) and polymeric methylene diphenyl diisocyanate (pMDI). Of particular interest was to investigate the effect of different types of chain extenders and their dosage on the degree of phase separation between hard and soft segments in polyurethane prepolymers. It has been well established that the stoichiometric ratio of isocyanate to hydroxyl (NCO/OH) and free isocyanate (NCO) content affects the properties of urethane prepolymers in terms of viscosity buildup, gelation onset, and curing performance [1,2,5,8,15,19,20,21,22,23,24]. In this work, the free NCO content was kept constant at 15 wt %, which is within the conventional industrially used range (14–18 wt %) [5]. Reaction conditions, such as temperature and the synthesis process, can also affect the performance of polyurethane adhesives [1,2,4,13,25,26]. In the absence of catalysts, the reaction temperature was carefully controlled and kept under 100 °C, in order to suppress the occurrence of side reactions like branching and cyclisation [27]. The effect of synthesis method, i.e., utilization of the one-stage versus the two-stage process, was also investigated.

## 2. Experimental

### 2.1. Materials

Polymeric methylene diphenyl diisocyanate (pMDI) (NCO% = 32.2, functionality = 2.3), polypropylene glycol 2000 (PPG 2000) (OH value = 56 mg KOH/g, 98%), di-propylene glycol (di-PPG) (99%), tri-propylene glycol (tri-PPG) (99%), butanediol (99%), pentanediol (99%), mono-isocyanate (99%) and tetrahydrofuran (THF) (99%) were all obtained from Sigma-Aldrich, Helsinki, Finland. The polyols and chain extenders were pre-dried at 60 °C using reduced pressure.

### 2.2. The Synthesis of Urethane Prepolymers

NCO-terminated chain extended urethane prepolymers were synthesized by polyaddition reactions of difunctional polyols and difunctional chain extenders with excess isocyanates. The synthesis reactions were carried out in a three-neck round bottom flask equipped with a mechanical stirrer, a dropping funnel, and a water condenser connected to a nitrogen inlet. ATR-FTIR was used to monitor the formation of urethane prepolymers, which will be elaborated in ATR-FTIR section.

#### 2.2.1. One Shot Process

Initially excess pMDI, PPG 2000 and chain extender were added into the reactor, and the system was flushed with nitrogen. An inert atmosphere was maintained throughout the whole process. The mixture was then heated to 80 °C and stirred vigorously at 800–1000 rpm. When the reaction had been completed, the mixture was cooled down to below 40 °C, and an excess of pMDI was added in order to reach a constant theoretical isocyanate content of 15 wt %. Eventually, 0.2 wt % of mono isocyanate was added as a moisture scavenger to enhance storage stability [28].

#### 2.2.2. Two-Stage Process

Excess pMDI was poured into the reactor and the system was preheated to 60 °C, and simultaneously, the reactor was flushed with nitrogen and an inert atmosphere was maintained throughout the duration of the reaction. In the first stage, PPG 2000 was dropped into the reactor at 60 °C under vigorous stirring of 800–1100 rpm. The temperature was raised to 80 °C. When the reaction between pMDI and PPG 2000 had been completed, the reactor was cooled down to 60 °C. In the second stage, chain extender was dropped in at 60 °C. When the reaction was completed, the mixture was cooled down. At a temperature below 40 °C, additional pMDI was added in order to reach a constant theoretical isocyanate content of 15 wt %. Eventually, 0.2 wt % of mono isocyanate was added as a moisture scavenger to enhance storage stability [28].

The urethane prepolymers were kept air-tight after production until curing started. The composition of urethane prepolymers is shown in Table 1. These urethane prepolymers were labeled as UP**X-***CE*-***Y***: UP refers to urethane prepolymer; **X** refers to synthesis processes (**1** refers to one-shot process, **2** refers to two-stage process); *CE* refers to the different types of chain extenders (*O* refers to no chain extender; di-PPG is abbreviated as *DI*; tri-PPG is abbreviated as *TRI*; pentanediol is abbreviated as *PEN*, butanediol is abbreviated as *BUT*); ***Y*** refers to the molar ratio between chain extender and PPG 2000.

The final NCO weight content (NCO-wt %) of all samples, measured by titration with *N*,*N*′-dibutylamine, was 15 ± 0.5 wt %, which is in good agreement with the theoretical value. The theoretical NCO-wt %, the molar ratio of NCO and OH (NCO/OH), and the weight ratio of hard segment (HS) and soft segment (SS) (HS/SS) were calculated according to Equations (1)–(3), respectively.

(1) $$\mathrm{NCO}\;\%=\frac{n\left(\mathrm{excess NCO}\right)\;\times \;M\left(\mathrm{NCO}\right)\;\times \;100\%}{\mathrm{mtot}}$$

(2) $$\frac{\mathrm{NCO}}{\mathrm{OH}}=\frac{n\left(\mathrm{pMDI}\right)\;\times \;\mathrm{functionality}}{n\left(\mathrm{polyols}\right)\;\times \;\mathrm{functionality}+n\left(\mathrm{diol}\right)\;\times \;2}$$

(3) $$\frac{\mathrm{HS}}{\mathrm{SS}}=\frac{\left(\mathrm{weight of}\;CE\;+\;\mathrm{weight of}\;\mathrm{pMDI}\right)\;\times \;100\%}{\mathrm{weight of polyols}}$$

In Equation (1), *n* (excess NCO) refers to mole of excess (free) isocyanate, and *M*(NCO) is the molar mass of isocyanate. In Equation (2), *n*(pMDI), *n*(polyols) and *n*(diol) refer to moles of pMDI, polyols and chain extenders, respectively.

As can been seen in Table 1, at a constant NCO weight content of 15 wt %, the HS/SS weight ratio increased and NCO/OH molar ratio decreased as a function of chain extender amount.

### 2.3. Characterization Methods

**ATR-FTIR** ATR-FTIR spectroscopy (Nicolet IS50 ATR-FTIR instrument, Thermo Fisher Scientific, Vantaa, Finland) was utilized to monitor the reaction progress and to characterize the final urethane prepolymers. Absorbance spectra were collected between 400 and 4000 cm^−1^ at a resolution of 4 cm^−1^. 

**NMR** The chemical structure of urethane prepolymers was examined by proton nuclear magnetic resonance (^1^H-NMR) spectroscopy, using a Bruker NMR 600 MHz spectrometer (Bruker, Coventry, United Kingdom). Deuterated chloroform (CDCl_3_) was used as a solvent.

**DSC** Thermal transitions of urethane prepolymers were measured by Q1000 DSC instrument (TA Instrument, Helsinki, Finland), over a temperature range of −90 to 80 °C at a heating/cooling rate of 10 °C/min. The first heating curve was analyzed.

**SEC** Prior to size exclusion chromatography (SEC) measurement, the urethane prepolymers were first treated with excess methanol to end-cap the free NCO groups [[5]]. Then excess methanol was removed, and the end-capped polymers were dried and prepared for SEC measurements. The molecular weight of sol polymers was measured using a SEC instrument (Shimadzu, Duisburg, Germany) with a low temperature evaporative light scattering detector (LT-ELSD, Sedere sedex 85LT, Sedere, Helsinki, Finland), equipped with an PSS SDV (styrene-divinylbenzene copolymer network) guard column (8 mm × 50 mm) and PSS SDV gel columns (500 Å: 8 mm × 300 mm; 10^5^ Å: 8 mm × 300 mm) ((PSS polymer standards service GmbH, Mainz, Germany)). The number-average molecular weight (Mn), weight-average molecular weight (Mw) and polydispersity index (PDI = Mw/Mn) were calculated relative to polystyrene standards (PSS polymer standards service GmbH, Mainz, Germany), ranging from 374 to 2,570,000 g/mol (Mp). THF was used as the eluent at a flow rate of 0.8 mL/min.

**Viscosity** The viscosity measurement was conducted prior to curing. Brookfield viscometer was utilized to measure the viscosity of urethane prepolymers at 20 °C. The ratio of spindle to speed was 5/20.

**Mechanical strength of adhesive bonding** The samples were cured under standardized conditions for 7 days (7d), whereby curing had been completed. 7d’s tensile bond strength of cured samples was measured by using a tensile shear strength instrument (Instron 3366, INSTRON, Danderyd, Sweden), according to the standard DIN EN 1465.

## 3. Results and Discussion

### 3.1. Characterization and Properties of Polyurethane Prepolymers

#### 3.1.1. NMR

**Figure 2:**
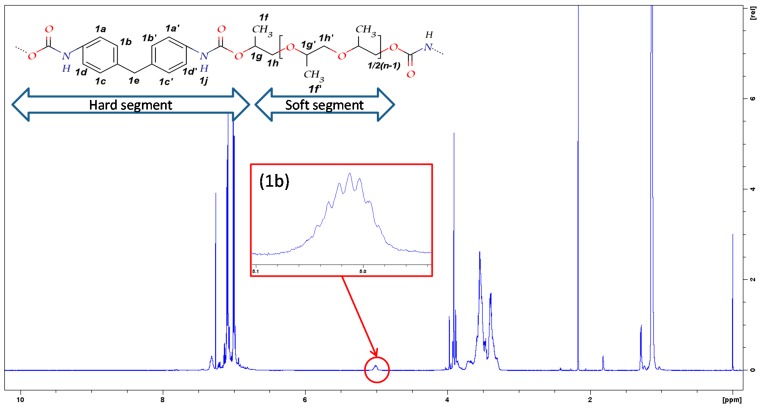
^1^H-NMR spectra of the blank sample UP**2**-*O*-***0***. The multiple peaks at 4.9–5.1 ppm range were enlarged and shown in Figure 2(1b).

**Figure 3:**
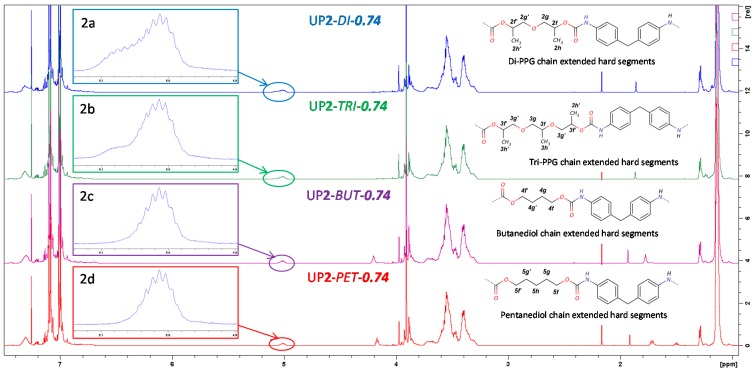
^1^H-NMR spectra of the chain extended samples UP**2**-*DI*-***0.74***, UP**2**-*TRI*-***0.74***, UP**2**-*PEN*-***0.74*** and UP**2**-*BUT*-***0.74***, and the sketched chemical structure for the chain extended hard segments. The multiple peaks at 4.9–5.1 ppm range were enlarged and shown in Figure 3(**2a**–**2d**).

The ^1^H-NMR spectra of the blank sample UP**2**-*O*-***0*** and the samples containing different chain extenders in the molar ratio of 0.74 are shown in Figure 2 and Figure 3, respectively. The characteristic peaks in these samples are assigned and listed in Table 2 and Table 3 [1,29]. The characteristic signal for N-H proton of allophanate at 10.85 ppm was not observed in ^1^H-NMR spectra [21,25,27]. The formation of urethane gave rise to a characteristic symmetric multiple peak at around 5 ppm (see Figure 2(1b)), which was assigned to PPG methine α to carbamate, as shown in Figure 2. Its intensity increased with urethane conversion.

**Figure 4:**
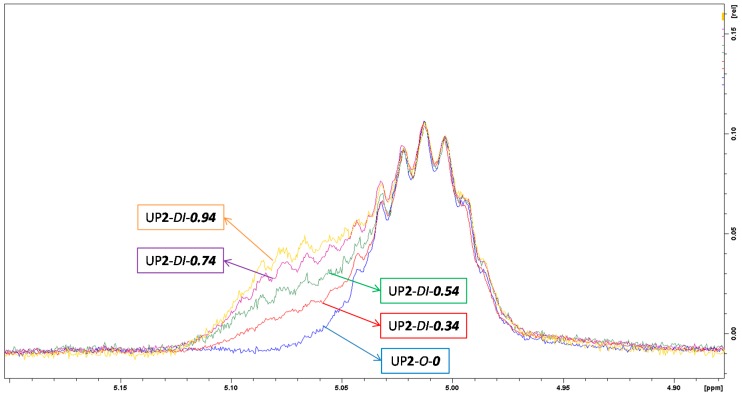
^1^H-NMR spectra of di-PPG chain extended samples UP**2**-*DI*-***0.34***, UP**2**-*DI*-***0.54***, UP**2**-*DI*-***0.74*** and UP**2**-*DI*-***0.94*** at a chemical shift range from 4.9 to 5.1 ppm.

Due to the similar chemical structures of repeating units of di- and tri-PPG within PPG 2000, the majority of peaks assigned to di- and tri-PPG chain extended hard segments overlapped with the main polymer peaks, except for resonances at 4.9–5.1 ppm. In contrast to UP**2**-*O*-***0*** (Figure 2(1b)), for UP**2**-*DI*-***0.74*** and UP**2**-*TRI*-***0.74*** (see Figure 3(2a,b)) shoulder multiple peaks at around 5.07 ppm were observed, which were assigned to chain extenders methines α to carbamate. By comparing the series of di-PPG chain extended samples and their chemical shifts at 4.9–5.1 ppm (Figure 4), the intensity of shoulder peaks at 5.07 ppm increased with the amount of di-PPG, which was attributed to the increasing formation of urethane linkages. Comparing Figure 3(2c, 2d) to Figure 2(1b), UP**2**-*PEN*-***0.74*** and UP**2**-*BUT*-***0.74*** showed multiple peaks at 4.9–5.1 ppm similar to UP**2**-*O*-***0***. Linear diols methylenes α to carbamate were assigned to 4.2 ppm. 

No significant differences between the ^1^H NMR spectra of samples prepared by one- or two-stage processes could be observed (not shown here). 

#### 3.1.2. Attenuated Total Reflectance Infrared Spectroscopy

During the synthesis of urethane prepolymers, intermediate and final polymers were analyzed by ATR-FTIR in order to monitor reaction conversions. This was accomplished by observing the changes of characteristic absorbance bands of different functional groups, such as free isocyanate and urethane groups. As the reaction between isocyanate and hydroxyl groups progressed, the intensity of the N=C=O stretching band at 2270 cm^−1^ decreased, and simultaneously, the intensity of the urethane C=O stretching peak at 1680–1760 cm^−1^ increased. The reaction was judged to be completed when no changes in the intensities of N=C=O and urethane C=O stretching peaks were recorded. The carbonyl stretching bands for urea analogues at 1625–1680 cm^−1^ were not found in final products, thus indicating that no significant side reactions between isocyanates with moisture had occurred during the preparation of urethane prepolymers.

**Figure 5:**
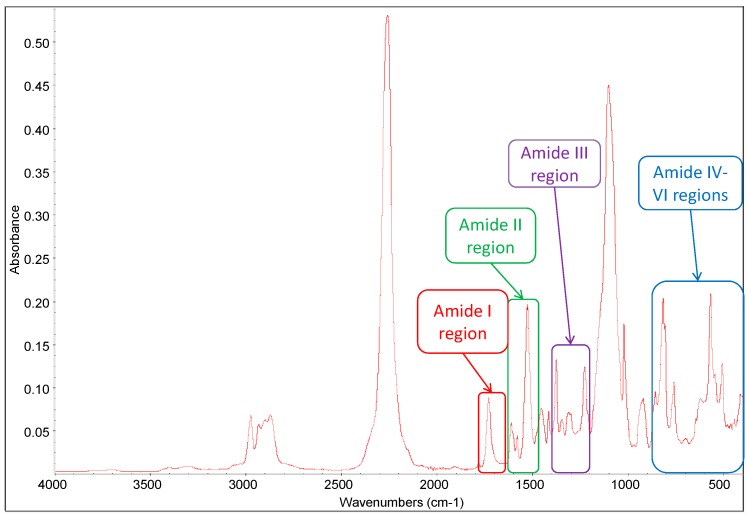
Attenuated total reflectance infrared spectroscopy (FTIR-ATR) spectrum of the blank sample UP**2**-*O*-***0***.

The ATR-FTIR spectrum of the blank sample UP**2**-*O*-***0*** is shown in Figure 5, and the assignments of its characteristic peaks are given in Table 4. In general, it has been earlier established that there are several characteristic amide absorptions in the FTIR spectrum of a polyurethane, including Amide I mode (1600–1800 cm^−1^: urethane C=O stretching, C–N stretching, and C–C–N deformation), Amide II mode (1500–1600 cm^−1^: N–H in-plane bending and the C–N stretching), Amide III mode (1200–1400 cm^−1^: C–N stretching vibration and NH deformations) and Amide IV-VI modes (400–800 cm^−1^: NH out-of-plane deformations) [14]. The urethane C=O stretching in the Amide I region and the bands in the Amide II region are sensitive to chain conformation and hydrogen bonding interactions, and the former is valuable for this type of polyurethane studies.

Since the driving force for the formation of dual phase structure in polyurethanes is related to hydrogen bonding interactions within or between the HS and SS, the organization of polymer chains and the extent of phase separation can be investigated through analyzing the patterns of hydrogen bonding interactions, which can be achieved by studying the urethane N**–**H stretching region (3200–3400 cm^−1^) and the C=O stretching region (Amide I: 1680–1760 cm^−1^) in FTIR spectra [11,14]. The hydrogen bonding associating with the urethane carbonyl group can be correlated with a shift in its stretching signals as follows: the non-hydrogen bonded carbonyl in the free urethane group is assigned to 1730–1760 cm^−1^; the hydrogen-bonded carbonyl group in the disordered (amorphous) conformation is assigned to 1700–1730 cm^−1^, which represents the hydrogen bonding between SS and HS; whereas the hydrogen bonded carbonyl group in ordered (crystalline) conformation is assigned to 1680–1700 cm^−1^, representing hydrogen bonding within HS [9,23,26]. Likewise, the free N–H (non-hydrogen bonded) and hydrogen bonded N–H absorbance bands can be assigned to 3400 cm^−1^ and −3300 cm^−1^, respectively [15,26].

**Figure 6:**
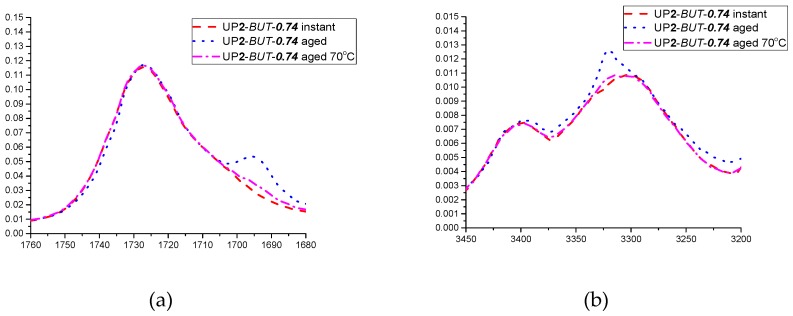
ATR-FTIR spectra at the C=O stretching band ranging 1680–1760cm^−1^ (**a**), and the N–H stretching band ranging 3200–3450 cm^−1^ (**b**): UP**2**-*BUT*-***0.74*** instant was measured soon after production; UP**2**-*BUT*-***0.74*** aged was measured after one month; UP**2**-*BUT*-***0.74*** aged 70 °C was measured after UP**2**-*BUT*-***0.74*** aged was reheated to 70 °C.

In addition, in order to study the effect of aging on the formation of hydrogen bonding, ATR spectra was also recorded at different time periods within one month. It was found that most samples did not show significant changes in the ATR-FTIR spectra upon aging, except for UP**1**/**2**-*PEN*-***0.74***, UP**1**/**2**-*PEN*-***0.94*** and UP**1**/**2**-*BUT*-***0.74***. All of the samples showed a shift of carbonyl peaks to lower wavenumbers, with an increasing intensity of peaks at 1680–1700 cm^−1^ and 3300 cm^−1^ within 1–2 days after production, which is closely related to their tendency for forming hydrogen bonding within HS. In particular, UP**1/2**-*BUT*-***0.74*** presented a strong shoulder peak at 1680–1700 cm^−1^ and a sharp peak at 3300 cm^−1^ after one day of aging, which indicated a higher content of highly ordered structures via substantial hydrogen bonding interactions within hard segments. These changes were not only time dependent, but also thermally reversible. A test was made with a one month-old UP**2**-*BUT*-***0.74***:UP**2**-*BUT*-***0.74*** that was reheated to 70 °C, with ATR-FTIR spectrum immediately recorded (labeled as UP**2**-*BUT*-***0.74*** aged 70 °C). The ATR spectra of UP**2**-*BUT*-***0.74*** at different stages (instant, aged and aged 70 °C) are compared in Figure 6, at the C=O stretching band ranging 1680–1760 cm^−1^ (left) and the N–H stretching band ranging 3200–3450 cm^−1^ (right). It was clearly observed that after the thermal treatment, the ATR-FTIR spectrum of UP**2**-*BUT*-***0.74*** aged 70 °C showed a significantly decreased intensity of peaks at 1680–1700 cm^−1^ and 3300 cm^−1^, which was almost identical to the initial UP**2**-*BUT*-***0.74*** spectrum. This is because increasing temperature weakened the hydrogen bonding within HS domains, and instead improved the phase mixing of HS and SS [15,26].

**Figure 7:**
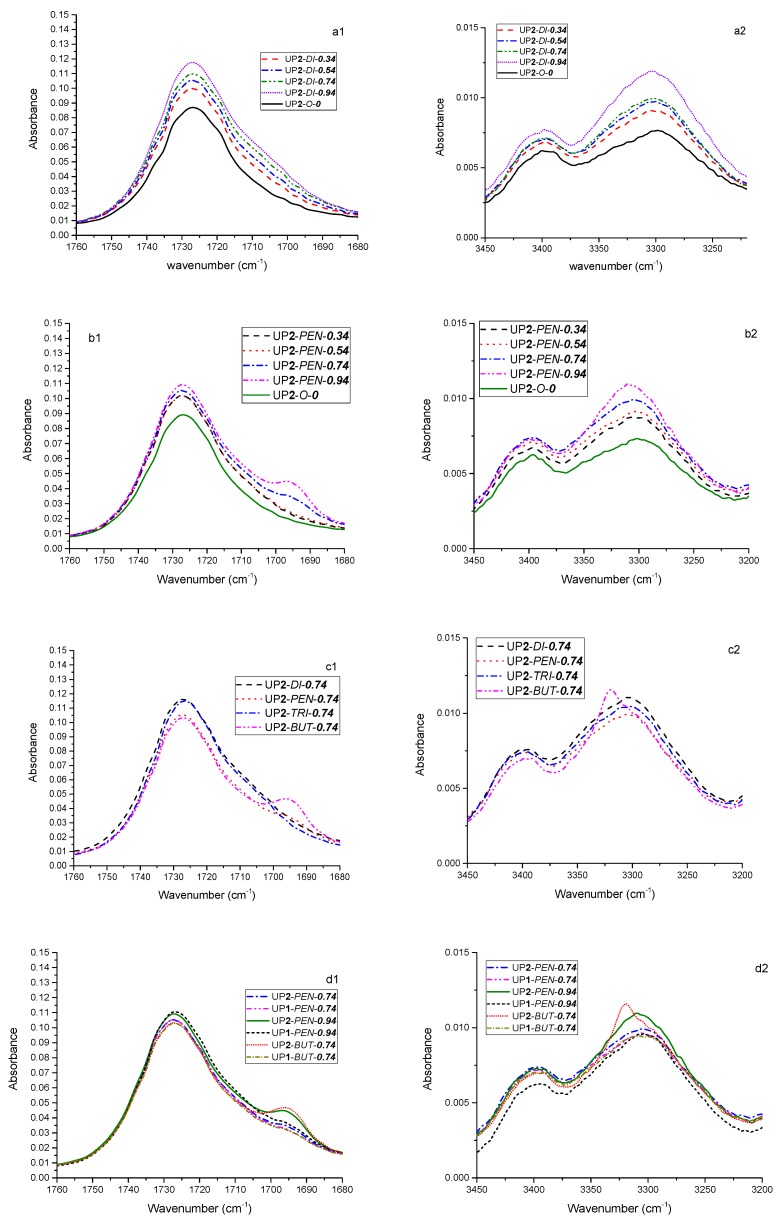
Comparison of ATR-FTIR spectra at the C=O stretching band ranging 1680–1760cm^−1^ (left) and N-H stretching band ranging 3200–3450 cm^−1^ (right): (**a1**,**a2**) samples prepared by the two-stage process with varying di-PPG amount compared with the blank sample UP**1**-*O*-***0***; (**b1**,**b2**) samples prepared by the two-stage process with varying pentanediol amount compared with the blank sample UP**1**-*O*-***0***; (**c1**,**c2**) samples prepared by two-stage process with 0.74 molar ratio of different chain extenders; (**d1**,**d2**) selected samples prepared by the two different synthesis processes.

Further ATR-FTIR spectra (C=O stretching band ranging 1680–1760 cm^−1^ and N–H stretching band ranging 3200–3450 cm^−1^) of one month aged samples are compared in Figure 7.

**Figure 8:**
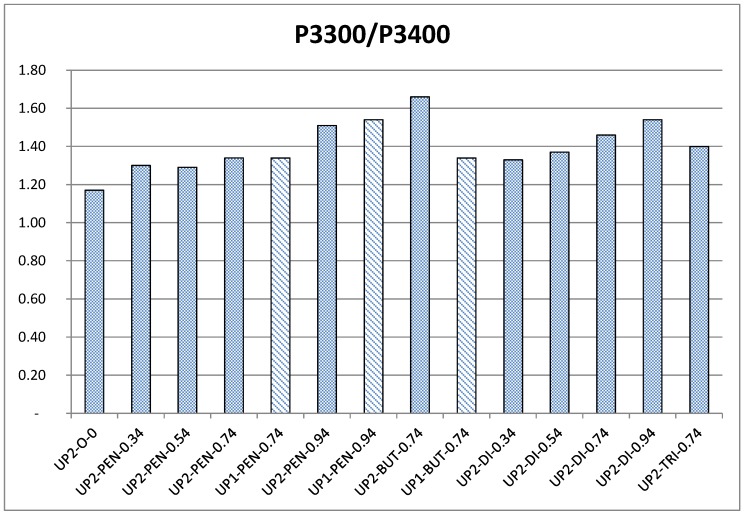
The intensity ratios of P3300 and P3400 of selected samples.

In order to quantify the contributions of chain extenders, the intensity ratios of the peak at 3300 cm^−1^ (P3300) and the peak at 3400 cm^−1^ (P3400) were calculated and shown in Figure 8.

According to Figure 7(a1,a2) and Figure 8, the intensity of peaks ranging between 1690 and 1720 cm^−1^ and the ratio of P3300/P3400 gradually increased as the amount of di-PPG increased. Particularly, the major change in the C=O stretching band was observed at 1700–1720 cm^−1^, which revealed that the majority of HSs formed disordered (amorphous) hydrogen bonding with the SSs. This is because the di-PPG contains branches that are capable of hindering the formation of highly ordered hydrogen bonding within HS. Furthermore, the structural similarity between di-PPG in HS and PPG 2000 in SS sufficiently enhanced miscibility between HS and SS [9]. Both could explain why the incorporation of di-PPG readily promoted the formation of disordered hydrogen bonding, and thus greatly contributed to phase mixing between HSs and SSs.

As can be seen from Figure 8, pentanediol had a more pronounced effect on the overall extent of hydrogen bond formation at higher dosages of 0.74 and 0.94 molar ratios. Moreover, it was generally observed in Figure 7(b1) that pentanediol chain extended samples showed different changes in C=O stretching bands in comparison to di-PPG, which indicated different patterns of hydrogen bonding. At a low dosage of pentanediol (0.34 to 0.54 molar ratio), the overall extent and type of hydrogen bonding were almost the same as in the case of di-PPG (Figure 8), where hydrogen bonding was primarily formed between HSs and SSs [Figure 7(b1)]. Whereas, at a high dosage of pentanediol (0.74 to 0.94), a shoulder peak corresponding to hydrogen bonded C=O absorbance in ordered conformation gradually appeared at 1680–1700 cm^−1^ and increased as a function of pentanediol amount, which reveals that the HSs associated into hard domains via hydrogen bonding. 

The influence of chain extender structures on the formation of hydrogen bonding was investigated by comparing samples containing different chain extenders in a molar ratio of 0.74, as shown in Figure 7(c1,c2) and Figure 8. In general, the overall extent of hydrogen bond was similar for all of these samples, except UP**2**-*BUT*-***0.74*** which presented a sharp peak shift to a higher wavenumber (~3320 cm^−1^), and an extremely high ratio of P3300/P3400. According to Figure 7(c1), UP**2**-*DI*-***0.74*** and UP**2**-*TRI*-***0.74*** had a tendency to form disordered hydrogen bonding interactions (1700–1720 cm^−1^), whereas UP**2**-*PEN*-***0.74*** and UP**2**-*BUT*-***0.74*** exhibited more pronounced, highly ordered hydrogen bonding interactions, as evidenced by the appearance of shoulder peaks at 1680–1700 cm^−1^). This peak was most intensive for UP**2**-*BUT*-***0.74***. Combined with the findings in the N–H stretching absorbance region (Figure 7(c2) and Figure 8), UP**2**-*BUT*-***0.74*** contained substantial amounts of highly ordered hydrogen-bonded structures. It has been earlier reported that linear chain extenders with an even number of CH_2_ (e.g., butanediol) readily promote HS association into hard domains via hydrogen bonding, and hence enhance phase separation in comparison to the one with an odd number of CH_2_ (e.g., pentanediol) [17,26].

The synthesis process has more influence on those samples that also otherwise tend to phase separate easily (UP**1/2**-*PEN*-***0.74***, UP**1/2**-*PEN*-***0.94*** and UP**1/2**-*BUT*-***0.74***). Thus, the ATR spectra of these samples were compared in Figure 7(d1,d2) and Figure 8. Based on Figure 8, the butanediol chain extended samples UP**1/2**-*BUT*-***0.74*** were the most susceptible to the influence of the synthesis process, the one-shot sample being much less hydrogen bonded than the two-stage sample. Figure 7(d1) gives additional support that both UP**1**-*BUT*-***0.74*** and UP**1**-*PEN*-***0.94*** (one-shot) showed less pronounced shoulder peak compared to their counterparts (two-stage). This finding confirms the statement that the one-shot process produce a urethane prepolymer with more randomly distributed polymer chains, and more dispersed HSs that eventually enhance phase mixing between the HS and SS [13,26].

#### 3.1.3. Appearance

The appearance of the urethane prepolymers in terms of color, transparency and viscosity can be correlated with the architecture and morphology of the polymers. For instance, phase separation between SSs and HSs can cause light scattering and thereby have an influence on the transparency [13]. The viscosity results are shown in Table 5.

The blank samples PU**1/2**-*O*-***0*** containing no chain extenders were clear, brownish liquids and were the least viscous (Table 5), and their appearance did not change during storage. The brownish color was attributed to the effect of pMDI. When chain extenders were added, the viscosity of urethane prepolymers dramatically increased as the amount of chain extender increased, because the increasing amount of hard segments intermolecularly associate via hydrogen bonding, and form physical cross-linking that restricts the mobility of soft segments [5,23].

**Figure 9:**
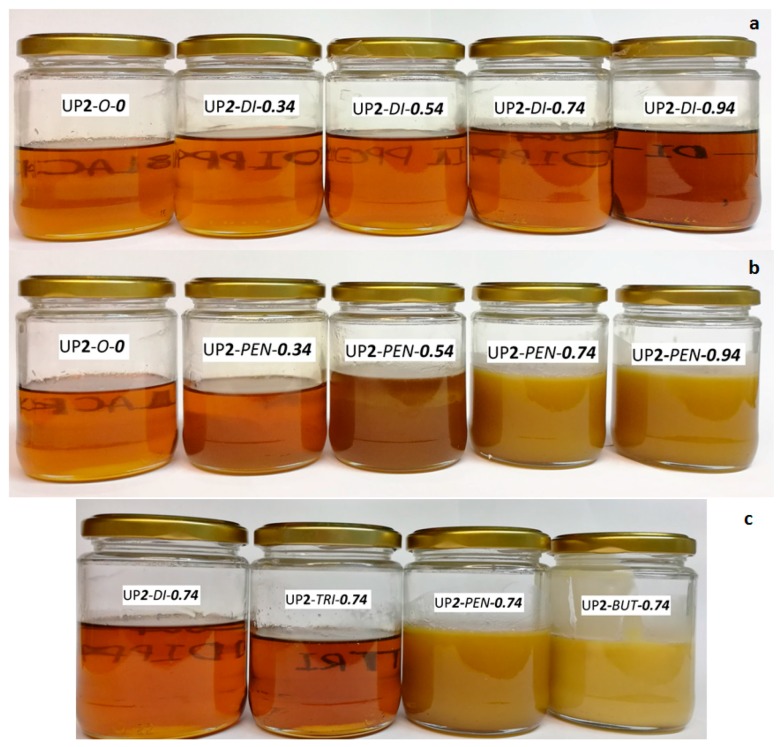
The appearance of samples made in two-stage processing: (**a**) di-PPG chain extended samples compared with the blank sample UP**1**-*O*-***0***; (**b**) pentanediol chain extended samples compared with the blank sample UP**1**-*O*-***0***; (**c**) samples with a 0.74 molar ratio of different chain extenders.

The type and amount of chain extenders had a significant influence on the appearance of samples. The appearance of two-stage prepared urethane prepolymers are shown in Figure 9. Similarly as blank samples, di-PPG chain extended urethane prepolymers were brownish and transparent (see Figure 9a). As the di-PPG amount increased, the color became deeper and the viscosity increased, which was attributed to increasing pMDI addition and HS content. On the contrary, urethane prepolymers containing linear chain extenders (butanediol and pentanediol) showed quite different behaviors. As can be seen in Figure 9b, the pentanediol chain extended urethane prepolymers showed a tendency towards lighter colors, gradual appearance changes from transparency to translucency (invisible marks), and increasing viscosity as the pentanediol amount increased, which was more pronounced at high pentanediol amounts (≥0.74). In particular, UP**1**/**2**-*BUT*-***0.74*** presented a completely opaque, yellow and waxy appearance (not flowing) (Figure 9c). These findings are consistent with the ATR-FTIR results: the incorporation of di-PPG and tri-PPG greatly contributed to phase mixing between HS and SS; in contrast, the incorporation of pentanediol and butanediol readily promoted the immiscibility between HS and SS, whereupon at higher chain extender ratios (≥0.74) prepolymers exhibited an opaque yellow appearance and extremely high viscosities. According to the viscosity results in Table 5, the effect of synthesis processes on viscosity is not fully straightforward, i.e., most samples prepared via the one-stage process had a lower viscosity than their two-stage counterparts but this was not the case for all samples. Nonetheless, the results clearly reveal that viscosity is significantly increased by the extent of highly ordered hydrogen bonding structures.

#### 3.1.4. Differential Scanning Calorimetry

The thermal transitions of all the urethane prepolymers at a temperature range from −90 to 80 °C are presented in Table 5. Since the chemical composition of SS is kept constant in all samples, the changes in thermal transitions of urethane prepolymers are closely linked to the HS chain length and the degree of phase separation (miscibility of HS and SS) [1,11,20]. The transparent samples (UP**1**/**2**-*O*-***0***, di-PPG series (UP**1**/**2**-*DI*-***0.34***/***0.54***/***0.74***/***0.94***), UP**1**/**2**-*TRI*-***0.74*** and UP**1**/**2**-*PEN*-***0.34***/***0.54***) showed only one distinct Tg within the analyzed temperature range that was higher than that of neat PPG 2000 (−70 °C), indicating extensive phase mixing of HS and SS [20]. The Tg of urethane prepolymers are primarily determined by the SS derived from long chain polyol, especially for the ones with low HS content. For instance, blank samples UP**1**/**2**-*O*-***0*** have the lowest and narrowest Tg. The chain extended urethane prepolymers follow the expected trend that Tg increases and becomes broader as chain extender amount increases, due to the greater interactions of increasing HSs that limit the movements of SSs.

Translucent and opaque samples (UP**1**/**2**-*PEN*-***0.74***/***0.94*** and UP**1**/**2**-*BUT*-***0.74***) exhibited both Tg and a melting peak within the analyzed temperature range, which indicated greater phase separation. The Tgs were mainly attributed to a phase of mixed HS and SS, while the melting peak derived from highly ordered hard domains. By comparing UP**1**/**2**-*PEN*-***0.74*** and UP**1**/**2**-*PEN*-***0.94***, it was generally noticed that Tg, Tm and enthalpy all slightly increased as the amount of pentandiol increased, indicating that the increasing formation of HS contributes to both disordered and highly ordered structures. UP**1**/**2**-*BUT*-***0.74*** showed the highest Tm and enthalpy, which confirmed that they have the most highly organized structures among these samples. These findings are in accordance with the previous results. The influence of the synthesis processes was not clearly detected in the Tg results (ΔTg ≤ ±2 °C).

#### 3.1.5. Size Exclusion Chromatography

Molecular weight measurement helps to gain an insight into the formation of urethane polymer chains, and particularly whether the chain extenders are incorporated via reactions with the isocyanate terminals of the urethane polymer chains, or with free pMDI. No significant differences in molecular weights between similar polymers synthesis by one- or two-stage processes could be found by SEC measurements. Therefore, data is only shown for polymers prepared by the two-stage process.

**Figure 10:**
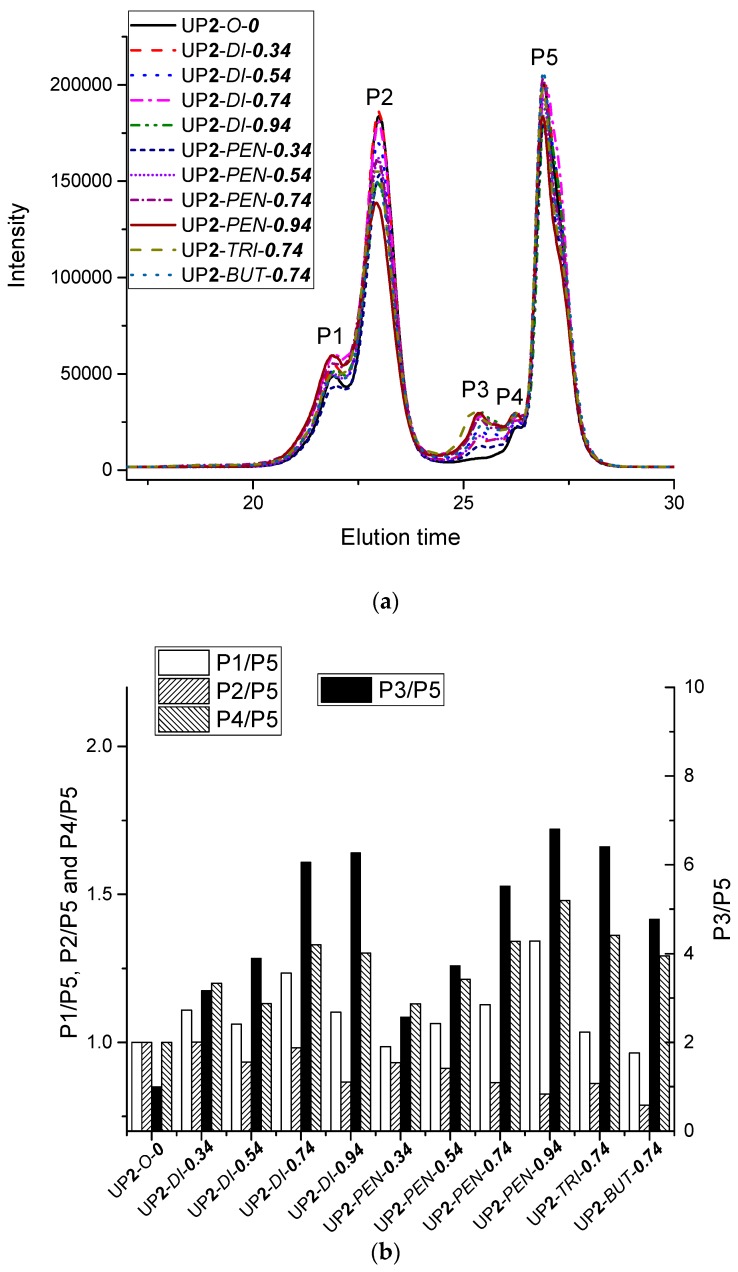
(**a**) Size exclusion chromatography (SEC) chromatograms of the samples made via the two-stage process; (**b**) The relative intensities of peaks P1/P5, P2/P5, P3/P5 and P4/P5 are compared to the blank sample UP**2**-*O*-***0***.

SEC chromatograms of the two-stage urethane prepolymers (Figure 10(a)) showed overlapping peaks in a range of elution times from 21 to 29 min, mainly consisting of five peaks: the peak P5 at the longest elution time (27 min) corresponded to the unreacted pMDI; the peaks P3 and P4 appearing at ca. 25.4 and 26 min were assigned to the hard segments consisting of pMDI that had been reacted with chain extenders; the major peak P2 at 23 min and the peak P1 at ca. 21.9 min represented the NCO-terminated urethane polymers.

According to the relative intensities of different peaks compared in Figure 10(b), it was generally observed that the majority of chain extenders contributed to P3, and its relative intensity increased with the chain extender amount, as chain extenders readily reacted to low molecular weight fractions like free pMDI. In addition, a minority of chain extenders contributed to P1 and P2 by reacting with the isocyanate terminals of polyurethane. The average molecular weight of the high molecular weight fraction was calculated for the combined peak P1 + P2. The obtained values for Mn, Mw and Mw/Mn are summarized in Table 6. The molecular weight and its distribution slightly increased as the chain extender amount increased. This trend was more evident for the samples with pentanediol than for the samples with di-PPG.

### 3.2. Properties of Cured Polyurethane Films

#### Bond Strength

Since the NCO content was constant, cured films were expected to contain similar amount of urea linkages. Thus, the bond strength of cured samples was more dependent on structural differences in the prepolymers: the content of hard segment, degree of hydrogen bonding, and phase separation. 

**Figure 11:**
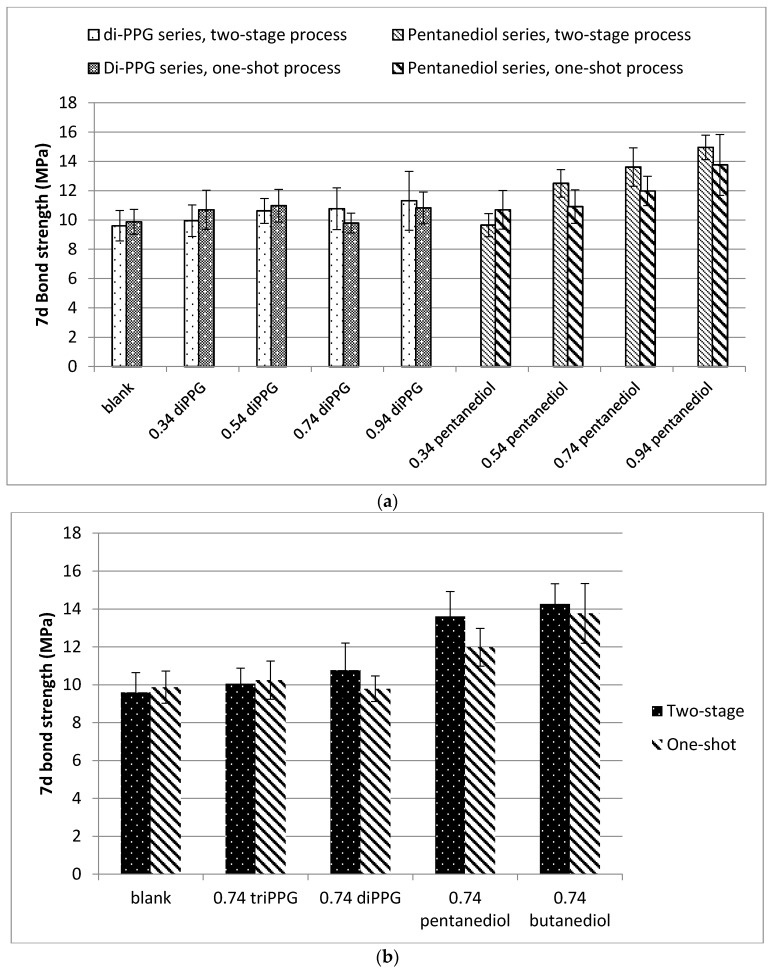
(**a**) The 7d bond strength of cured samples containing different amounts of di-PPG and pentanediol, prepared both by one-shot and two-stage process; (**b**) The 7d bond strength of cured samples with 0.74 molar ratio of different chain extenders, prepared in one-shot and two-stage processes.

Figure 11a shows the 7d bond strength results of cured samples containing different amounts of di-PPG and pentanediol. The trend was clearly towards higher bond strengths with increasing amounts of chain extender. As free NCO weight content was constant, the increase in bond strength was attributed to increasing HS content and hydrogen bonding interactions. This trend was more obvious for the cured samples containing pentanediol, as pentanediol chain extended samples readily form highly ordered physical-cross-linked regions within the HS that reinforce the material. As expected, the cured samples prepared by two-stage process showed a more pronounced trend than their one-shot counterparts, as two-stage synthesis resulted in more organized polymer chain conformations.

Figure 11b exhibits the influence of different chain extenders on bond strength of the cured samples at a constant molar ratio of 0.74. Not surprisingly, the cured samples containing butanediol or pentanediol exhibited the highest bond strength, due to strong hydrogen bonding interactions and a higher extent of phase separation. However, as these prepolymers also showed high viscosities and turned wax-like after a couple of days of storage, they did not fulfill the initial goal of high strength combined with industrially acceptable flow properties. In case of tri-PPG and di-PPG, in turn, the enhancement in strength was relatively low compared to the blank sample.

Finally, in this series of samples, the optimal combination of properties was achieved using pentanediol chain extender at a molar ratio of 0.54. For the UP**2**-*PEN*-***0.54***, over 12 MPa lap shear strength accompanied by low viscosity was obtained (Table 5). Clearly enhanced lap shear strength was also reached using di-PPG at a CE molar ratio of 0.94.

## 4. Conclusions

A series of chain extended urethane prepolymers was synthesized, and the effects of different chain extenders, as well as the influence of the synthesis process were systematically investigated. As anticipated, the incorporation of chain extenders enhanced the adhesive strength of MCPUs, and this enhancement was dependent on the structure of chain extenders and the synthesis process. The one-shot process produced urethane prepolymers with more randomly organized polymer chains than the two-stage process, leading to better phase mixing. Compared to the linear chain extenders, the branched chain extenders (di-PPG and tri-PPG) generated less phase segregation between HS and SS, enabling low viscosity. Compared to non-chain extended polyurethane, clearly enhanced lap shear strength was reached only when using di-PPG at a high molar ratio of 0.94. On the contrary, the incorporation of linear chain extenders readily promoted HS association into hard domains via hydrogen bonding interactions within HS. The degree of phase separation was particularly pronounced for butanediol, and/or when the linear chain extender molar ratio was no less than 0.74. However, phase separation significantly increased the viscosity, whereby at a high linear chain extender amount (≥0.74 molar ratio), the urethane prepolymers became wax-like (no flow) and were therefore not ideal for their intended use. Above all, polyurethane with a 0.54 molar ratio of pentanediol showed strong adhesive strength with simultaneously low viscosity. 

This work demonstrates that chain extenders can be used to prepare one-component polyurethane adhesives that exhibit simultaneously low viscosity and enhanced shear strength after curing. The type and the amount of CE, together with the synthesis manner determine the degree of HS hydrogen bonding, and thereby the degree of phase segregation. This in turn is the main contributing factor both for viscosity and shear strength build-up.

## Figures and Tables

**Table 1:** The compositions of urethane prepolymers.

Sample (UPX-*CE*-*Y*) *	pMDI:PPG2000:CE molar ratio	NCO/OH molar ratio	HS/SS
UP**1**-*O*-***0***	7.44:1:0	8.57	1.12
UP**2**-*O*-***0***			
UP**1**-*DI*-***0.34***	8.08:1:0.34	6.94	1.23
UP**2**-*DI*-***0.34***			
UP**1**-*DI*-***0.54***	8.46:1:0.54	6.33	1.29
UP**2**-*DI*-***0.54***			
UP**1**-*DI*-***0.74***	8.84:1:0.74	5.85	1.36
UP**2**-*DI*-***0.74***			
UP**1**-*DI*-***0.94***	9.22:1:0.94	5.47	1.42
UP**2**-*DI*-***0.94***			
UP**1**-*PEN*-***0.34***	8.08:1:0.34	6.94	1.23
UP**2**-*PEN*-***0.34***			
UP**1**-*PEN*-***0.54***	8.46:1:0.54	6.33	1.29
UP**2**-*PEN*-***0.54***			
UP**1**-*PEN*-***0.74***	8.84:1:0.74	5.85	1.36
UP**2**-*PEN*-***0.74***			
UP**1**-*PEN*-***0.94***	9.22:1:0.94	5.47	1.42
UP**2**-*PEN*-***0.94***			
UP**1**-*TRI*-***0.74***	8.84:1:0.74	5.85	1.36
UP**2**-*TRI*-***0.74***			
UP**1**-*BUT*-***0.74***	8.84:1:0.74	5.85	1.36
UP**2**-*BUT*-***0.74***			

* **X**: synthesis processes: **1**—one-shot process, **2**—two-stage process; *CE*: chain extender: *DI—*di-PPG, *TRI*—tri-PPG, *PEN*—pentanediol, *BUT*—butanediol; ***Y***: the molar ratio of chain extender to PPG 2000.

**Table 2:** Proton chemical shifts of the blank sample UP**2**-*O*-***0****.*

Segment	Assignment of protons	Shift (δ, ppm)
Hard	1a–1d and 1a′–1d′	6.9–7.1
	1e	3.9–4.0
	1j	7.3
Soft	1f′	1.1–1.2
	1f	1.3
	1g	5.0
	1g′, 1h, 1h′	3.2–3.8

**Table 3:** Proton chemical shifts for the chain extended hard segments of samples UP**2**-*DI*-***0.74***, UP**2**-*TRI*-***0.74***, UP**2**-*PEN*-***0.74*** and UP**2**-*BUT*-***0.74.***

Sample Code	Assignment of protons	Shift (δ, ppm)
UP**2**-*DI*-***0.74***	2h, 2h′	1.3
	2f, 2f′	5.0
	2g, 2g′	3.2–3.8
UP**2**-*TRI*-***0.74***	3h, 3h′	1.3
	3f′	5.0
	3f, 3g, 3g′	3.2–3.8
UP**2**-*BUT*-***0.74***	4f, 4f′	4.2
	4g, 4g′	1.8
UP**2**-*PEN*-***0.74***	5h	1.5
	5g, 5g′	1.7
	5f, 5f′	4.2

**Table 4:** FTIR-ATR characteristic peaks assignment for UP**2**-*O*-***0***.

Functional group	Wavenumber/cm^−1^
pMDI N=C=O stretching	2270
pMDI aromatic C=C stretching	1611, 1578
Urethane prepolymer (UP) urethane N–H stretching	3200–3400
UP urethane C=O stretching	1680–1760
UP urethane N–H bending and C–N stretching	1524–1527
UP urethane C–O stretching	1220–1222
UP aromatic C=C stretching	1598
PPG 2000 C–O–C stretching	1100
PPG 2000 CH_2_ stretching vibration (symmetric and asymmetric)	2857–2940

**Table 5:** Differential scanning calorimetry (DSC) results and viscosity of urethane prepolymers.

Sample (UPX-*CE*-*Y*) *	DSC results			Viscosity
	Tg_1_ of 1st heating	Tm of 1st heating	Melting enthalpy	
UP**1**-*O*-***0***	−48.58	--	--	3640
UP**2**-*O*-***0***	−50.01	--	--	3640
UP**1**-*DI*-***0.34***	−45.98	--	--	6500
UP**2**-*DI*-***0.34***	−46.27	--	--	6060
UP**1**-*DI*-***0.54***	−44.68	--	--	6150
UP**2**-*DI*-***0.54***	−43.67	--	--	7740
UP**1**-*DI*-***0.74***	−43.82	--	--	8600
UP**2**-*DI*-***0.74***	−42.93	--	--	11,080
UP**1**-*DI*-***0.94***	−39.63	--	--	22,400
UP**2**-*DI*-***0.94***	−39.05	--	--	12,000
UP**1**-*PEN*-***0.34***	−48.15	--	--	6920
UP**2**-*PEN*-***0.34***	−47.43	--	--	5100
UP**1**-*PEN*-***0.54***	−44.83	--	--	9220
UP**2**-*PEN*-***0.54***	−44.54	--	--	6820
UP**1**-*PEN*-***0.74***	−43.10	59.98	2.176	17,500
UP**2**-*PEN*-***0.74***	−45.41	53.25	1.688	45,600
UP**1**-*PEN*-***0.94***	−43.82	58.28	2.138	50,880
UP**2**-*PEN*-***0.94***	−42.52	56.52	2.304	52,600
UP**1**-*TRI*-***0.74***	−38.91	--	--	9950
UP**2**-*TRI*-***0.74***	−40.35	--	--	9600
UP**1**-*BUT*-***0.74***	−43.10	83.58	3.016	36,200
UP**2**-*BUT*-***0.74***	−43.53	81.41	3.229	44,000

* **X**: synthesis processes: **1**—one-shot process, **2**—two-stage process; *CE*: chain extender: *DI*—di-PPG, *TRI*—tri-PPG, *PEN*—pentanediol, *BUT*—butanediol; ***Y***: the molar ratio of chain extender to PPG 2000.

**Table 6:** The integrated molecular weight and distribution for P1 and P2.

Sample (UPX-*CE*-*Y*) *	Total average of P1 and P2		
	Mn	Mw	Mw/Mn
UP**2**-*O*-***0***	4330	5250	1.21
UP**2**-*DI*-***0.34***	4430	5450	1.23
UP**2**-*DI*-***0.54***	4500	5570	1.24
UP**2**-*DI*-***0.74***	4560	5710	1.25
UP**2**-*DI*-***0.94***	4540	5630	1.24
UP**2**-*PEN*-***0.34***	4380	5320	1.22
UP**2**-*PEN*-***0.54***	4480	5600	1.25
UP**2**-*PEN*-***0.74***	4540	5740	1.27
UP**2**-*PEN*-***0.94***	4760	6090	1.28
UP**2**-*TRI*-***0.74***	4480	5580	1.25
UP**2**-*BUT*-***0.74***	4460	5490	1.23

* **X**: synthesis processes: **2**—two-stage process; *CE*: chain extender: *DI*—di-PPG, *TRI*—tri-PPG, *PEN*—pentanediol, *BUT*—butanediol; ***Y***: the molar ratio of chain extender to PPG 2000.
